# Assessing the Impact of the Rapid Response Team Training Program on the Performance Outcomes of First-Year Post-Graduate Residents During Rapid Response Codes

**DOI:** 10.7759/cureus.83519

**Published:** 2025-05-05

**Authors:** Hong Thoai Nguyen, Nita Lohala, Surendra Sigdel, Bibek Adhikari, Salma Zeb, Daria Kotsar, Aamir Abdullah, Alvaro F Matos Arana, Yanira V Lagos, Ebram Said

**Affiliations:** 1 Internal Medicine, Ascension Saint Joseph - Chicago, Chicago, USA

**Keywords:** internal medicine residents, intern doctors, quality improvement and patient safety, quality improvement projects, rapid response team (rrt)

## Abstract

The transition to residency training in the United States presents distinct challenges for both American Medical Graduates (AMGs) and International Medical Graduates (IMGs), particularly in the context of the rapid response team (RRT) involvement. This study investigates the effect of a structured educational intervention entitled "Enhancing the Rapid Response Team's Understanding" on the performance of postgraduate year-one (PGY-1) Internal Medicine residents at the Ascension Saint Joseph Hospital, Chicago. The intervention aimed to enhance the theoretical knowledge and practical competencies related to RRT activation, fostering better integration into multidisciplinary teams during acute clinical deteriorations. A total of 29 PGY-1 residents participated. Five pre-intervention and four post-intervention responses were received, with both assessments evaluating knowledge acquisition, task performance, and confidence levels. The intervention involved didactic sessions on RRT protocols, followed by supervised inpatient floor rotations. The pre- and post-intervention results indicated a significant improvement in task performance, with a 45.83% increase in mean scores (from 48 to 70), though this change did not reach statistical significance (p=0.1096). Despite an increase in the number of tasks performed by participants (p=0.0397), no significant differences were observed in overall knowledge, awareness (p=0.119), or confidence (p=0.722) regarding the RRTs. Feedback from the participants highlighted the intervention's value, with 75% rating the sessions as important and of good quality. While the small sample size limited statistical power, the findings suggest that structured educational interventions for interns may positively impact their ability to perform critical tasks during RRT activations. Further research with larger cohorts is needed to comprehensively assess the long-term effects of such interventions on clinical preparedness and patient care outcomes.

## Introduction

The transition to residency training in the United States represents a critical and often demanding period for both American Medical Graduates (AMGs) and International Medical Graduates (IMGs) [1]. During this initial phase, interns must rapidly translate theoretical knowledge into effective clinical decision-making within a high-acuity, fast-paced healthcare setting. A key responsibility that interns are expected to assume early in their training is active participation in the rapid response teams (RRTs), which serve as frontline responders for events of clinical deterioration. Engagement in RRTs not only leads to a need to change clinical judgment and the way procedures are performed but also provides a valuable platform for experiential learning and development of leadership competencies essential for progression to more senior roles in the subsequent years of training. For the IMGs in particular, this transition is further complicated by the need to acclimate to the structure and culture of the U.S. healthcare system, which may differ substantially from their prior training environments, particularly in institutions where RRTs are not a standard component of inpatient care. These compounded stressors highlight the unique and multifactorial challenges interns face during the early stages of the U.S. graduate medical education [2].

This study aims to assess the impact of a structured educational intervention, “Enhancing the Rapid Response Team's Understanding,” on the performance of postgraduate year one (PGY-1) Internal Medicine residents during RRT activations. The course was designed to improve the theoretical knowledge and the practical competencies of the interns, which are pertinent to the execution of Rapid Response Codes, thereby facilitating their effective integration into the multidisciplinary RRT. The primary objective is to determine whether targeted preparatory training enhances clinical preparedness, situational confidence, and progressive autonomy in managing acute events of patient deterioration. This investigation was conducted at the Ascension Saint Joseph - Chicago, a hospital in Chicago, and evaluated pre- and post-intervention metrics, focusing on the acquisition of critical decision-making skills and procedural proficiency relevant to RRT participation.

## Materials and methods

Study design

This prospective study was conducted over six months from July 2024 to December 2024. As part of the study protocol, senior residents were assigned to develop and deliver a series of educational presentations focused on the structure, function, and clinical application of the RRT. These sessions were designed to provide incoming interns with practical and case-based instruction to enhance their understanding and integration into the RRT system.

Study population

The study cohort comprised 29 PGY-1 internal medicine trainees, including categorical, transitional year, and preliminary residents, enrolled in an internal medicine residency program at Ascension Saint Joseph - Chicago, a mid-tier academic community hospital located in the Midwestern U.S.

Recruitment and ethical considerations

Participation in the study was determined by the availability and willingness of the interns. Prospective participants were notified of the study via a private communication platform and individual outreach. The selection of interns was based on self-reported availability and voluntary interest in participation. Participants accessed the survey through a hyperlink and completed it anonymously. No identifiable personal information was collected. As a result, their identities remain unknown to the investigators. Given the anonymous nature of data collection and absence of personally identifiable information, formal written informed consent was deemed unnecessary in accordance with ethical guidelines for minimal-risk research. The study protocol was reviewed and approved by the Institutional Review Board (IRB) of Ascension Health (Approval Numbers: RIL20240035 and MOD00007649).

Pre-intervention assessment

Participants completed a pre-intervention assessment comprising multiple-choice items designed to evaluate baseline knowledge and clinical understanding of Rapid Response Codes. Responses were collected via Google Forms (Google, California, US) over a secure, encrypted online platform. Demographic variables, including age, sex, and prior clinical experience, were also obtained for analysis.

Intervention

Following the completion of the pre-intervention assessments, a series of didactic sessions focused on RRT protocols were integrated into the Morning Reports and Noon Conferences. These educational interventions were specifically designed to enhance the clinical preparedness of the incoming interns in managing Rapid Response activations. The curriculum covered evidence-based approaches to common acute clinical scenarios frequently encountered during RRT events, including but not limited to hypotension, falls, tachyarrhythmias, bradyarrhythmias, hypoxic episodes, and acute chest pain. Upon conclusion of the didactic component, interns transitioned to inpatient floor rotations, where they were expected to apply these principles in direct patient care under supervision.

Post-intervention assessment

Six months after the intervention, participants were administered a follow-up survey comprising multiple-choice items analogous to those included in the pre-intervention assessment, with the addition of items specifically designed to evaluate the perceived quality of the RRT training. This post-intervention instrument aimed to assess changes in the knowledge and clinical competencies of the participants attributable to the educational intervention. Survey responses were collected via via Google Forms (Google, California, US) over a secure data collection platform. 

Statistical analysis

Both pre- and post-intervention responses were collated on Google Sheets (Google, California, US) for analysis. Continuous variables were described using means, medians, and standard deviations, while categorical variables were expressed as percentages. Comparisons of pre- and post-intervention continuous data were conducted using Welch’s t-test to assess the statistical significance of changes in knowledge scores. Differences in categorical proportions before and after the intervention were evaluated using Fisher’s exact test. A two-tailed p-value of ≤0.05 was considered statistically significant. Data preprocessing, visualization, and statistical analyses were conducted using IBM SPSS Statistics for Windows, Version 29 (Released 2023; IBM Corp., Armonk, New York, United States).

Data confidentiality and protection

Participant data were collected anonymously and securely stored, with access limited exclusively to authorized research personnel. All electronic records were maintained on password-protected computers and encrypted storage devices to ensure data confidentiality and integrity.

## Results

**Figure 1:**
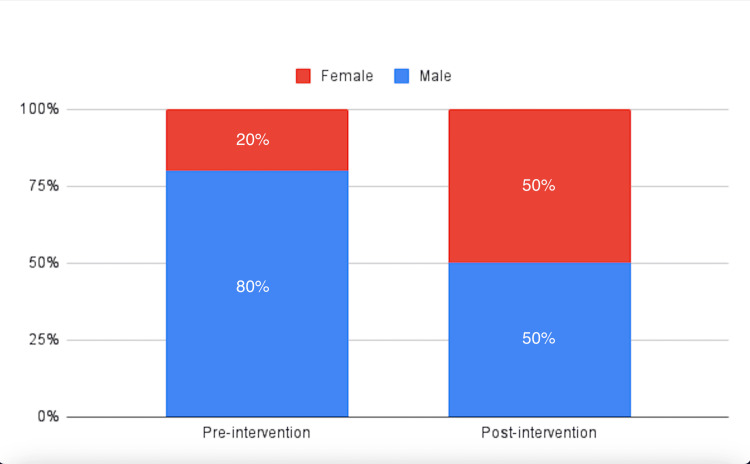
Gender distribution of the participants in the pre- and post-intervention survey

Out of the 29 new residents, five (20% female) participated in the pre-intervention survey, and four (50% female) (Figure 1) participated in the post-intervention survey.

The pre-intervention results indicated a mean score of 48, a median score of 40, and a standard deviation (SD) of 22.8 (Table 1).

**Table 1:** Descriptive statistics

	Mean	Median	Standard deviation
Pre-intervention score	48	40	22.8
Post-intervention score	70	70	11.55

**Figure 2:**
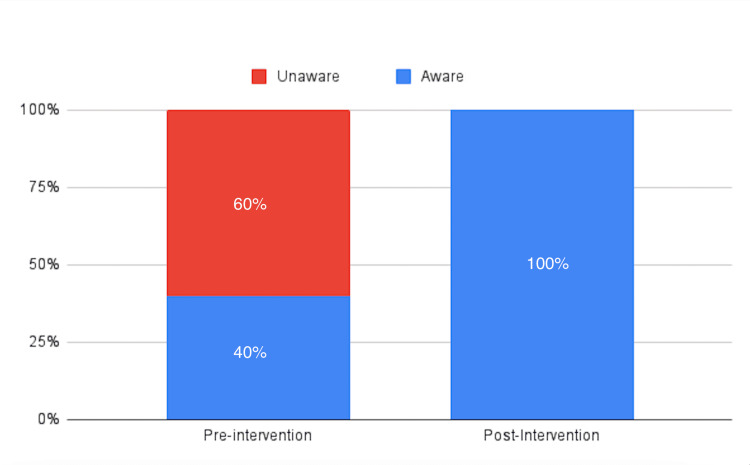
Pre- and post-intervention awareness proportions among the participants

In terms of awareness, 40% of participants were familiar with the RRT, while 60% were not (Figure 2).

**Figure 3:**
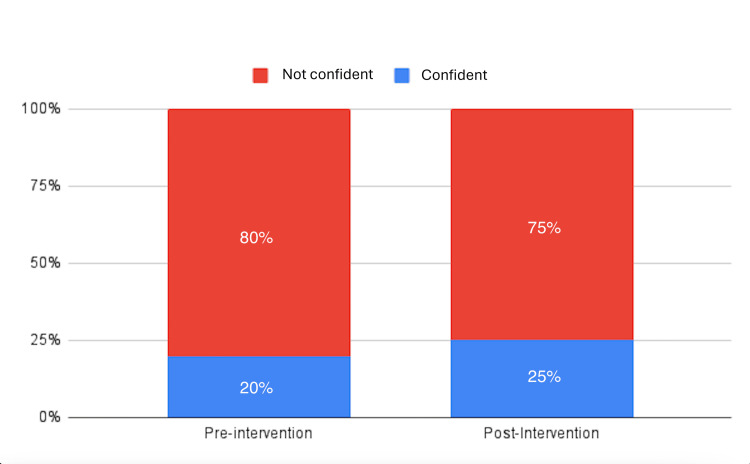
Confidence levels of the participants pre- and post-intervention

Regarding confidence, 20% of participants expressed confidence while attending the RRT, while 80% reported feeling a lack of confidence (Figure 3).

**Figure 4:**
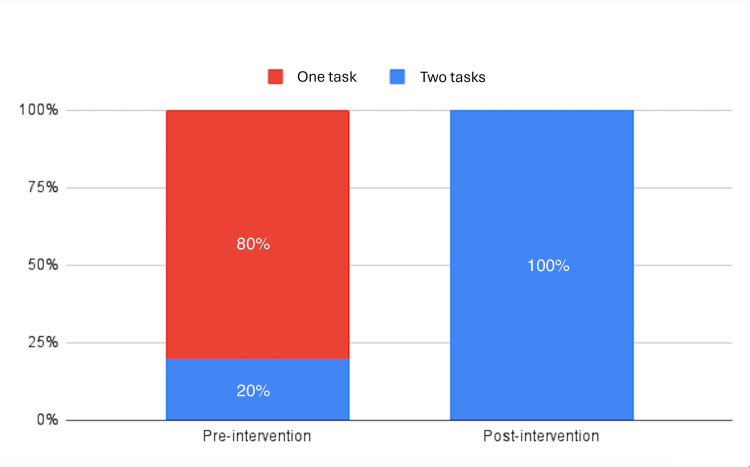
Proportion of participants performing tasks pre- and post-intervention

When asked about task performance during Rapid Response Codes, 80% of participants were able to perform one task, and 20% could perform two tasks (Figure 4).

Post-intervention results showed an increased mean score of 70, a median score of 70, and a reduced SD of 11.55 (Table 1). The mean score demonstrated an increase of 45.83% post-intervention. Additionally, 100% of participants were aware of the RRT after the intervention (Figure 2). In terms of confidence, 25% of participants felt confident during RRT attendance, while 75% were not confident (Figure 3). Moreover, 100% of the participants could perform two tasks in the Rapid Response Codes (Figure 4).

Regarding the quality of the RRT presentations, 75% of the participants rated the presentation as important, while 25% expressed a neutral opinion. Furthermore, 75% found the quality of the presentation to be good and applicable, whereas 25% considered it neutral and inapplicable. Half of the participants felt that the presentation met most of their expectations, while the other half believed it met only some of them. When asked about future presentations, 50% of the participants requested content on how to run codes, and 25% expressed interest in learning more about the Advanced Cardiac Life Support (ACLS) rhythm protocols.

Considering the small sample sizes (n1 = 5 for pre-intervention, n2 = 4 for post-intervention), a Welch’s t-test was conducted to assess the differences in means between the pre- and post-intervention groups. The resulting p-value was 0.1096, (t ≈ -1.88, df ≈ 6), which is greater than the conventional significance threshold of 0.05, indicating no statistically significant difference between the means of the two groups.

Fisher’s exact test was used to evaluate differences in the proportion of participants aware of the RRT between the pre- and post-intervention groups. The p-value obtained was approximately 0.119, which is greater than 0.05, suggesting no statistically significant difference in awareness proportions between the two groups.

A similar analysis using Fisher’s exact test was also conducted to assess differences in confidence levels between the pre- and post-intervention groups. The p-value was approximately 0.722, which exceeds 0.05, indicating no significant difference in the confidence levels.

Finally, the same test was applied to determine whether there was a significant difference in the number of tasks participants could perform between the pre- and post-intervention groups. The p-value was approximately 0.0397, which is less than 0.05, indicating a statistically significant difference in task performance between the two groups. In a two-tailed test, the p-value would be approximately 0.0794 (0.0397 × 2), which is just above the 0.05 threshold. However, the one-tailed result aligns with the directional implication of the data.

## Discussion

An RRT is a multidisciplinary group of clinicians responsible for providing immediate care to hospital patients exhibiting objective or subjective signs of clinical deterioration. A Rapid Response System (RRS) represents a comprehensive hospital-wide strategy aimed at enhancing the identification and management of deteriorating or at-risk patients. The RRS consists of an afferent limb (comprising the criteria and mechanism for activation), an efferent limb (the RRT itself), an administrative limb (responsible for the daily coordination of the team), and a quality improvement or governance limb [3].

RRTs have been shown to reduce the incidence of in-hospital cardiac arrests [4-6], lower hospital mortality rates [4,7,8], and improve end-of-life care practices [9]. Other reported benefits from a single-center study include a decrease in adverse events following major surgery [10]. Additionally, RRTs may facilitate expedited ICU transfers, enabling ward staff to devote more time to other patients. Emerging data suggest that it may be possible to predict which patients will require ICU admission following RRT activation [11].

In the U. S. and globally, medical graduates begin their residency programs in July each year [1]. For new interns to transition smoothly into effective team members of an RRT, they must be adequately prepared and familiar with the process of RRT activation, leadership, and the management of deteriorating patients. Moreover, attending RRTs provides new interns with critical opportunities to gain medical knowledge and clinical skills, fostering their confidence in assuming code leadership roles as they progress to senior levels of training, particularly in the categorical Internal Medicine residency.

This study involved 29 new interns, including categorical, preliminary, and transitional year residents. The mean scores of pre- and post-intervention assessments were 48 and 70, respectively, with a p-value of 0.1096 (>0.05), suggesting no statistically significant difference between the two time points. Despite a 45.83% increase in the mean score post-intervention, the results did not reach statistical significance. Furthermore, there was no statistically significant difference in the awareness of RRTs, with a p-value of 0.119 (>0.05). Similarly, no significant change in confidence levels was observed (p-value of 0.722 (>0.05)).

However, a statistically significant difference was found in the number of tasks performed, with a p-value of 0.0397 (<0.05). For a two-tailed test, the p-value would be approximately 0.0794 (0.0397 × 2), which exceeds 0.05, though the one-tailed result supports the directional hypothesis of the data.

The study was conducted anonymously and voluntarily, with only five interns participating pre-intervention and four responding post-intervention. The small sample size limited the study's power. Although no significant improvements in awareness or preparedness regarding RRTs were observed based on mean score analysis, the significant increase in the number of tasks performed by new interns remains a noteworthy finding. It is anticipated that this will enhance the confidence of the interns and support their development into effective team leaders in the future.

Regarding the intervention’s perceived value, 75% of participants considered the RRT presentation important, with good quality and applicability. Fifty percent reported that the presentation met most of their expectations, while the other half felt it met only some of them. Requests for additional presentations on code leadership and ACLS rhythm protocols were made by 50% and 25% of the participants, respectively. These findings suggest several avenues for future research on this topic.

Given this study's limitations, further investigations with larger sample sizes are needed to better assess the impact of such interventions on intern preparedness and awareness, which could ultimately improve the quality of care provided by new interns. Further similar studies should include objective performance data in their study structure. There is a lack of comparable studies employing similar models, which underscores the need for additional research in this area. However, we found a pilot study that assessed a team-based, interdisciplinary RRT training program for first-year residents to enhance readiness before leading RRTs in their second year. The program improved test scores, self-reported confidence, and team performance in simulated RRT scenarios. These findings suggest that structured RRT training can enhance resident education, team dynamics, and decision-making skills, potentially improving patient outcomes [12]. Another study evaluated the impact of high-fidelity simulation (HFS) on the self-confidence of new nurses and their activation of the RRT in a Level I trauma center. Findings showed that HFS immediately and significantly improved self-confidence, and it was sustained till five months after training. However, the effect on RRT activation varied across units, suggesting other factors may influence real-world application [13].

## Conclusions

In conclusion, this study demonstrates the potential value of structured educational interventions in enhancing the participation of PGY-1 Internal Medicine residents in RRT activations. While no significant improvements were observed in the overall knowledge scores, awareness, or confidence, there was a noteworthy increase in task performance, suggesting enhanced practical competencies. This finding underscores the importance of targeted training to improve the ability of interns to respond effectively during events of clinical deterioration. Participant feedback indicated that the intervention was well-received, with many requesting additional training on code leadership and ACLS protocols. Although the small sample size limited the statistical power, these results highlight the potential for educational interventions to support the growth and preparedness of interns in high-acuity situations. Further research with larger cohorts is needed to better understand the long-term impact of such programs and refine residency training to improve clinical outcomes in acute care settings.
